# Design and evaluation of a new nurse-led case management intervention for bariatric surgery patients

**DOI:** 10.1016/j.mex.2023.102049

**Published:** 2023-02-02

**Authors:** Cláudia Amaro Santos, Manuel Carvalho, João Gregório

**Affiliations:** a Hospital Espírito Santo de Évora, EPE, Évora, Portugal; b CRI.COM – Centro Responsabilidade Integrada de Cirurgia da Obesidade e Metabólica, Évora, Portugal; c CBIOS – Universidade Lusófona's Research Center for Biosciences & Health Technologies Lisbon, Portugal

**Keywords:** Case manager, Perioperative care, Bariatric surgery, Nurse case management, e-health, Design Science Research Methodology

## Abstract

This paper provides a new management about the treatment of obesity, that have a growing incidence worldwide. A management centered on the patients’ needs during the bariatric surgery is necessary. In this context, patient monitoring and follow-up by a case manager, who guides the provision of specialized care focused on patient's adaptation to the new reality, can prove to be essential to achieve better outcomes. This study, guided by the Design Science Research Methodology (DSRM), will have as main objective to design a new intervention (Case-managing program) aimed at patients undergoing bariatric surgery. As secondary objectives, we intend to analyze the influence of the new intervention in the perioperative period and impact on several clinical and humanistic endpoints. In the evaluation phase, an experimental, controlled, and randomized study (RCT) will be developed, with an intervention group (IG) and a control group (CG). The CG will receive the usual care and the IG, will receive the intervention for an expected period of one year. This project aims to be the first study to investigate the effect of a long-term specialized case-management intervention (face-to-face and e-health) in patients who are candidates for bariatric surgery during all the perioperative periods. This method presents:

• The results will be reported the patients related outcomes measures for bariatric surgery

• The results are expected to provide an overview of the most effective case management interventions for long-term better results on bariatric surgery

• Allowing researchers to design and propose a new case management for bariatric surgery

## Graphical abstract

**Figure:**
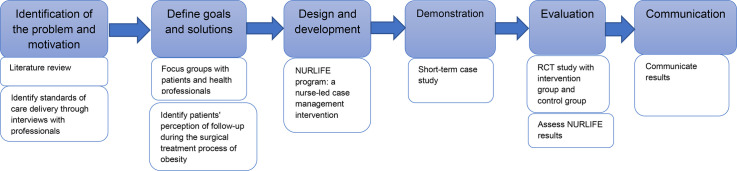

Specifications table

**Table:** 

Subject area	Medicine and Dentistry

More specific subject area:	*Case manager; bariatric surgery; nurse-led; patient-centered care; e-health*
Name of your method:	*Design Science Research Methodology*
Name and reference of original method:	*- Hevner AR, March ST, Park J, Ram S. Design Science in Information Systems Research. MIS Q. 2004;28(1):75–105.* *- Gregório J, Reis L, Peyroteo M, Maia M, Mira da Silva M, Lapão LV. The role of Design Science Research Methodology in developing pharmacy eHealth services. Res Soc Adm Pharm RSAP. December 2021;17(12):2089–96.*
Resource availability:	*No data are used in this study*

## Overview

### Research question

Does a specialized case-management intervention for patients who are candidates for bariatric surgery have an impact on post-surgical weight regain? How can this intervention contribute to lifestyles’ changes, in increasing well-being and self-care, and in the functional adaptation of patients in the perioperative period?

### State of the art

Obesity is considered an epidemic of the 21st century, with more than 4 million deaths worldwide [1]. It manifests itself as a chronic disease, but also as a risk factor for other pathologies and consequently worse health outcomes. The latest statistics from the World Health Organization (WHO) show that 1.9 billion adults are overweight, and more than 650 million adults are obese, with a body mass index (BMI) greater than 40 kg/m2. Obesity high prevalence demands a high priority intervention [2].

Bariatric surgery is considered the best long-term treatment for severe obesity and its comorbidities [3]. In addition to weight loss, bariatric surgery improves quality of life and self-esteem, allowing patients to improve their lifestyle and consequently maintain their long-term results [4]. Treating obesity induces a remission of diabetes in more than 50% of patients, in addition to preventing diabetes development [5,6]. International guidelines suggest that bariatric surgery indication should begin with a BMI greater than 40Kg/m2 or a BMI greater than 35Kg/m2 with associated comorbidities [7].

Despite being the most effective treatment for obesity, it is not a cure for obesity, and awareness of this fact is important for the success of treatment. This success depends on several factors. One of them is the preparation for surgery, usually with multidisciplinary orientation, focusing on health promotion, well-being and self-care, functional readaptation, and pre-qualification, in line with patient satisfaction [8]. In this process, the perioperative period is particularly important. This period can be divided in two phases: pre-surgery (or preoperative) and post-surgery (or postoperative).

Preoperative interventions usually focus on lifestyle changes, nutritional education, promotion of physical activity and cognitive behavioral therapy. Many times, these interventions achieve some weight loss, thus contributing to fewer surgical complications during the postoperative phase [9]. Prior contact with the person allows establishing an empathic relationship with the patient and family, assessing their perceptions and expectations, educating and teaching. The use of educational strategies, with organized and systematized information before bariatric surgery, encourages an attitude of self-care and lifestyle changes [10], thus empowering patients with knowledge that allows them to have an active and participatory attitude in their process of functional readaptation and adoption of healthy lifestyles [11].

There are discrepancies identified between the expectations of professionals and patients about the surgical treatment of obesity. Even among professionals there are differences regarding the importance given to the surgery outcomes. Professionals are more concerned with weight and comorbidities, while patients place greater importance on quality of life and well-being in general [12]. Also, evidence suggests that some patients are demotivated and disappointed during the process because they had unrealistic perspectives of weight loss [13].

In the follow-up and monitoring of patients with other chronic conditions, “case manager” nurses or “nurse-led case-management” interventions improved the health of chronic disease patients, with improved health outcomes [14]. The figure of these specialized professionals induces quality of care and reduces health costs [15]. Very recently, with the COVID-19 pandemic, the role of a case manager nurse in the follow-up of diabetic patients brought a significant improvement in the management of the disease and in the reduction of complications [16].

Achieving the best outcomes of bariatric surgery in the long-term depends on how patients experience, understand and accept the changes that develop in the perioperative period. It is in this context that the “nurse-led case-management” interventions may allow a better management of the surgical process.

The figure of case management in health is not exclusively performed or assumed by nurses. However, in chronic diseases, nurses have usually been the figure chosen in different contexts [17]. This fact is often due to their comprehensive vision of human needs and self-care promotion, as well as evaluating and planning care. Nurse case management focuses on achieving objectives through a dynamic nursing process, with a theoretical framework, which allows for humanistic care, through in-depth knowledge of the patient's biopsychosocial context, providing joint decision-making with other professionals in care planning [18].

Behavioral change is important in bariatric surgery for the maintenance of long-term results, which suggests that it is important that interventions for behavioral change in bariatric surgery patients begin before surgery and continue in the extended postoperative period. This complete, coordinated, and comprehensive long-term follow-up can be achieved through different forms of communication, namely using e-health, in a perspective of continuity and proximity to patients, in a holistic approach to the bariatric patient, based on a relationship with empathy, respect, and patient involvement in all care decisions [20], [21], [22], [23].

Patients value how they have access and continuity to health care with the team, whether by phone (telemedicine) or in person (face-to-face), as well as the interpersonal relationship established between nurse and patient, in addition to the perception of greater support from health professionals [19]. Accessibility and the type of relationship between the patient and the case manager is a strong point for the health system, as the interventions carried out consider the goals and needs of each person, providing coordinated and continuous care. In bariatric surgery, the activities performed by the nurse case manager have the potential to improve health and allow better outcomes, reducing care costs and improving the quality of care.

### Objectives

Supported by DSRM, the main objective of this project is to design, implement and evaluate a new case-managing intervention for patients undergoing surgical treatment of obesity, to optimize and maintain the results of bariatric surgery.

## Methodology

This research project will follow a mixed methods approach, guided by the DSRM methodology that will allow us to design a new specialized “nurse-led case-management” intervention (Table 1).

**Table 1:** Design Science Research Methodology Cycle.

DSRM Activity	Design Science Research Methodology cycle	
	Objectives	Method/Tasks
Activity 1	Identification of the problem and motivation	Systematic review of the literature
Activity 2	Set objectives for the intervention	Focus Groups
Activity 3	Design and development of the NURLIFE program	In-depth analysis of the previous results Iterative process with health professionals and Information Technology developers
Activity 4	Demonstration of NURLIFE intervention	Case study
Activity 5	Evaluation the results of the NURLIFE	RCT study, with intervention groups and control groups, lasting approximately 14 months
Activity 6	Communication of results	Communicate the results of all development stages and activities

The DSRM is a methodology that has already been defined and tested in the healthcare setting [24,25]. A DSRM process should abide to guidelines that indicate which steps to follow, as well as their sequence and criteria for the next phase. Six stages are defined, starting with the diagnosis and identification of the problem, understanding previous research and practices, followed by the definition of objectives for a solution, the design and development, and the evaluation and subsequent communication of the results [25]. The main outcomes of this process are solutions, or artifacts [26], which can be of various types [27], that must possess two important characteristics: they must have purpose and novelty in solving a problem (purpose) in an innovative way (novelty) [24].

The project is to be developed in a Public Hospital of the Portuguese National Health Service, whose funding is provided by the State Budge. The health system in Portugal is constituted by a publicly funded National Health Service (NHS - Beveridge type), and by other sub-systems (mostly Bismarck type, supported by employers and workers contributions). People can also have a voluntary health insurance, which facilitates access to private providers. The public, private and social sectors that integrate the Portuguese health system act according to the principle of cooperation, guiding their activities by rules of transparency and prevention of conflicts of interest [28]. Public hospitals and Primary Health care centers work in cooperation through the newly implemented Local Units of Health (LUS), whose aim is to set strategic health goals in specific regions and coordinate care provision. One of the benefits of these LUS is the streamlining of the referral procedures from primary to secondary and tertiary care, facilitating patients’ access to the health system [29].

## Study design

### Activity 1

#### Objective - Problem identification and motivation

To know the latest evidence of perioperative follow-up of patients who are candidates for bariatric surgery, a systematic review of the literature will be carried out to identify the characteristics of interventions aimed at these patients, their typology, and respective results.

### Activity 2

#### Objective - Define objectives for the intervention

For this activity, we will use a qualitative approach with the focus group method. Three focus groups are expected to be held with representatives of the various stakeholders involved (patients and health professionals), to identify the objectives for this new intervention. These objectives are expected to be related to the set of endpoints (primary and secondary) of interest that will serve to measure the success or failure of the intervention in the evaluation activity. The focus group will have a previous script that will be prepared considering the results of activity 1. For further thematic analysis, the focus group will be recorded, and the topics analyzed by the research team to inform the next activity. Since it is important to know the context in which the intervention is carried out, the focus groups will be carried out with patients already enrolled in the perioperative phase, in a hospital in the south of Portugal. Besides patients, nutrition, psychology, nursing, and surgery professionals, who are part of the follow-up protocol of these patients, are expected to participate in one of the focus groups.

### Activity 3

#### Objective - Design and development of the NURLIFE program (Fig. 1)

In this activity, the “nurse-led case-management” intervention program will be developed to improve the management of the bariatric surgery process by patients in collaboration with the health team. This activity aims to determine the set of procedures and their temporal sequence, the team's skill mix, the flow of patients by the different elements of the team and the duration of the program. This intervention program (NURLIFE) is expected to be an e-health program, supported by face-to-face consultations. Behavioral changes will predictably be one of the main outcomes, providing patients with the tools to cope and manage the physiological changes that result from the process of bariatric surgery. Thus, the primary focus of the program is expected to be health education and motivation for lifestyle changes, the promotion of healthy lifestyles and the promotion of physical activity, aiming to improve anthropometric data and metabolic risk factors, as well as psychological support.

**Fig. 1:**
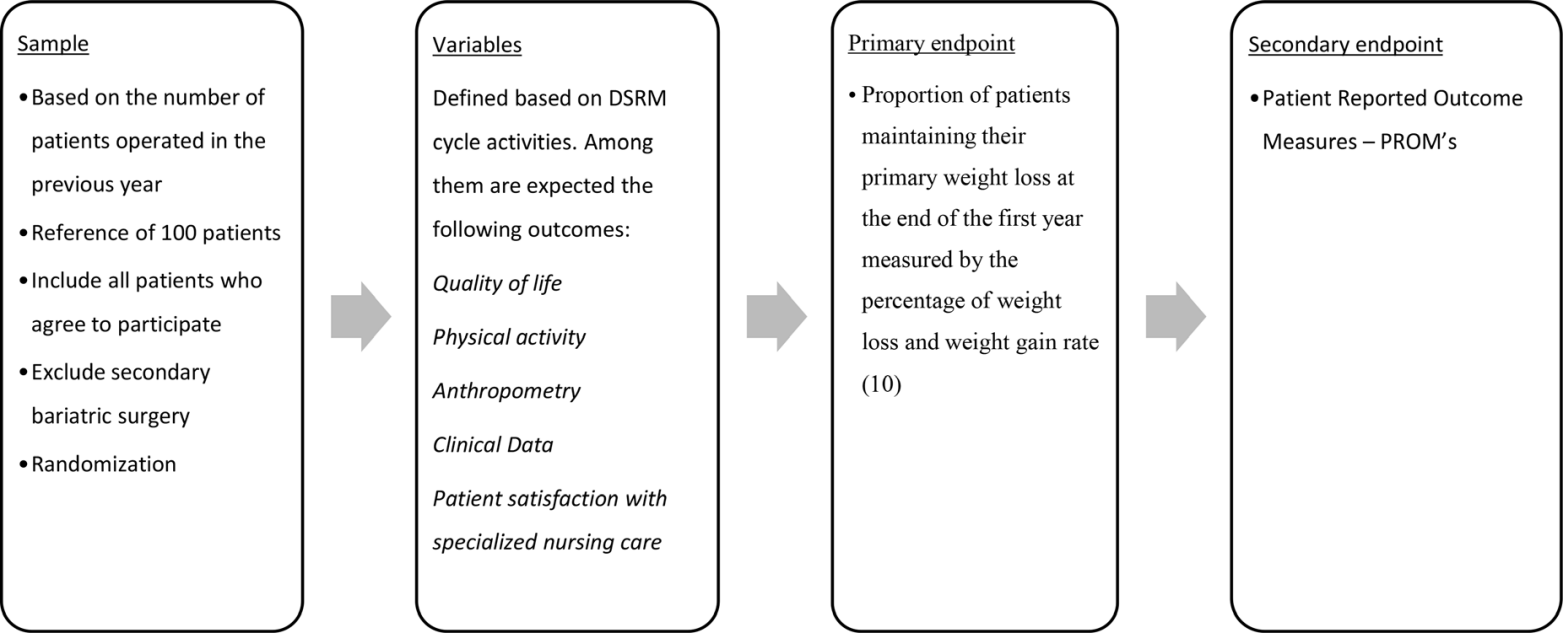
NURLIFE RCT Methodology.

### Activity 4

#### Objective - demonstration of the NURLIFE intervention

For the pursuit of this activity, a short-term case-control study is planned. The objective of this study will be to test the intervention designed in the previous step with a small number of participants, to optimize the intervention, collecting information from the participants (patients and multidisciplinary team) on the points to be improved. Likewise, the endpoints of interest will be measured, proceeding with their analysis to inform the sample calculation and power analysis necessary for the next activity.

### Activity 5

#### Objective: to evaluate the results of the NURLIFE program

To carry out this activity, a randomized clinical trial is planned, dividing the participants into two groups. The population of interest is the individuals on the list for surgical intervention (LIC) at the Integrated Responsibility Center for Surgery for Obesity and Metabolic Diseases, Hospital Espírito Santo de Évora, EPE, with criteria for bariatric surgery. The invitation to participate will be made in the context of a consultation and individuals who accept to participate in the study will be given an informed consent form, previously approved by the Ethics Committee of Universidade Lusófona and Hospital Espírito Santo de Évora, EPE. Participants will be randomly divided into a Control Group (CG) and Intervention Group (IG), starting the program upon enrollment of patients for surgery, approximately two months before surgery and ending 12 months after surgery. The GI will receive the NURLIFE intervention while the control group will receive routine care. Given the nature of the intervention, it is not possible to blind the participants.

### Activity 6

#### Communication

The Communication activity aims to disseminate the knowledge about the work developed throughout the project, with the submission of articles to journals in the area, communications at conferences and other meetings.

## Program features

The NURLIFE program is expected to have a maximum of 5 face-to-face moments, where the assessment instruments will be applied, and 7 non-face-to-face moments by tele or video consultation, for the intervention group, divided by the different specialties.

**Fig. 2:**
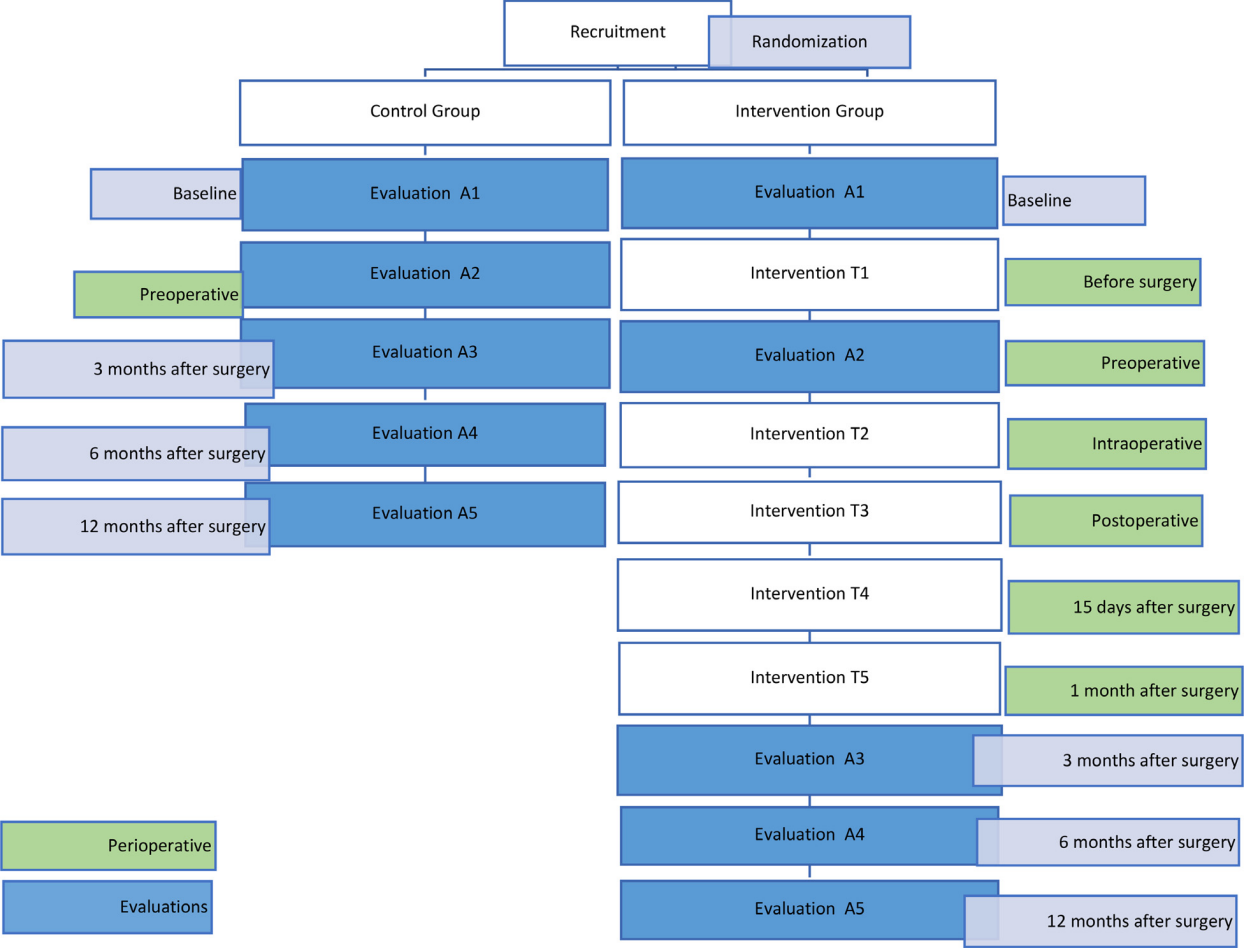
Prediction of the evolution and recruitment plan of the RCT study to evaluate the intervention.

The first contact with the participants will be at the first evaluation appointment, where the patient is enrolled for surgery, at the baseline moment (A1). At this moment, and after acceptance of informed consent, randomization is performed, the IG will start the intervention protocol and the patients in the CG will continue with their usual care. Patients in the CG will have face-to-face assessments, before surgery (A2) and three months (A3) after surgery, at the end of the perioperative period, and finally, six months (A4) and twelve months (A5) after surgery. The IG will have the same face-to-face assessments as the GC and will also have a teleconsultation one month before surgery (T1), face-to-face consultation in the intraoperative period (T2), in the immediate postoperative period (T3), in the mediate postoperative period, fifteen days after the surgery, they will have a teleconsultation (T4), another one month after the surgery, in the late postoperative period (T5), that will be the last teleconsultation (Fig. 2).

## Conclusion

This project aims to be the first study to investigate the effect of specialized interventions on patients who are candidates for bariatric surgery. It is expected that this project will provide evidence of the impact of mixed, face-to-face, and e-health programs, on the long-term maintenance of bariatric surgery results.

## Ethics statements

Each participant will be randomly assigned to each group after signing the informed consent and performing the initial assessments. All data collected will be identified with an identification ID, safeguarding the confidentiality of the data collected. Since the health personnel involved is shared by both IG and CG, the Hawthorne-effect can not be fully avoided when health professionals report subjective data. However, all the patients’ clinical quantitative data will be collected by health professionals that are subjected to ethical codes of practice, avoiding misreported data. A monitoring panel will be implemented, constituted by the surgical unit clinical director, the board director of the research center and an expert in clinical trials.

The study has the approval of both ethics committees from the academic institution (CE.ECTS – 06/22) and from the hospital (CE.HESE – 62/22).

## CRediT authorship contribution statement

**Cláudia Amaro Santos:** Conceptualization, Methodology, Writing – original draft. **Manuel Carvalho:** . **João Gregório:** Supervision, Methodology, Writing – review & editing.

## Declaration of Competing Interests

The authors declare that they have no known competing financial interests or personal relationships that could have appeared to influence the work reported in this paper.

## Data Availability

No data was used for the research described in the article.
